# Knockdown of zif268 in the Posterior Dorsolateral Striatum Does Not Enduringly Disrupt a Response Memory of a Rewarded T-Maze Task

**DOI:** 10.1016/j.neuroscience.2017.07.014

**Published:** 2018-02-01

**Authors:** Emma N. Cahill, George H. Vousden, Marc T.J. Exton-McGuinness, Ian R.C. Beh, Casey B. Swerner, Matej Macak, Sameera Abas, Cameron C. Cole, Brian F. Kelleher, Barry J. Everitt, Amy L. Milton

**Affiliations:** a Department of Psychology, University of Cambridge, CB2 3EB, UK; b Behavioural and Clinical Neuroscience Institute, Cambridge CB2 3EB, UK; c School of Psychology, University of Birmingham, B15 2TT, UK

**Keywords:** aDLS, anterior dorsolateral striatum, aDMS, anterior dorsomedial striatum, ANOVA, analysis of variance, A-O, ‘action-outcome’, BLA, Basolateral Amygdala, IEG, Immediate Early Genes, NAc, Nucleus Accumbens, ODN, Oligodeoxynucleotides, pDLS, posterior dorsolateral striatum, pDMS, posterior dorsomedial striatum, S-R, ‘stimulus-response’, Zif268, Zinc Finger Protein 225, zif268, memory, striatum, amygdala, hippocampus, T-Maze

## Abstract

Under certain conditions pavlovian memories undergo reconsolidation, whereby the reactivated memory can be disrupted by manipulations such as knockdown of *zif268*. For instrumental memories, reconsolidation disruption is less well established. Our previous, preliminary data identified that there was an increase in Zif268 in the posterior dorsolateral striatum (pDLS) after expression of an instrumental habit-like 'response' memory, but not an instrumental goal-directed 'place' memory on a T-maze task. Here, the requirement for Zif268 in the reconsolidation of a response memory was tested by knockdown of Zif268, using antisense oligodeoxynucleotide infusion into the pDLS, at memory reactivation. Zif268 knockdown reduced response memory expression 72H, but not 7d later. Western blotting revealed a non-significant increase in Zif268 in the pDLS in rats using response memories, but there was no change in Zif268 expression in the hippocampus following retrieval of a place memory. Zif268 expression increased in the basolateral amygdala after memory reactivation whether a response or place strategy was used during reactivation. We propose that Zif268 expression in the basolateral amygdala may be linked to prediction error, generated by the absence of reward at reactivation. Taken together, these results suggest a complex role for Zif268 in the maintenance of instrumental memories.

## Highlights

• Zif268 expression in the pDLS is not required for retrieval of habit-like memory.
• Knockdown of pDLS Zif268 expression reduces habit-like memory restabilization.
• Zif268 expression increased in the BLA after reward memory retrieval.
• Despite extended T-Maze training rats did not use a S-R strategy.

## Introduction

Habits are an adaptive way of performing behaviors with the minimum level of cognitive effort. However *compulsive* habits, e.g. in drug addiction, are highly maladaptive. For this reason, there has been great interest in developing treatments that allow compulsive habits to be overcome once established. One such treatment would disrupt the reconsolidation of habit memories so restoring control over behavior by the values of goals (Milton and Everitt, 2012).

Reconsolidation is the process by which memories become destabilized at reactivation, and subsequently updated or strengthened (Nader et al., 2000). Reconsolidation can be disrupted by antisense oligodeoxynucleotides (ASO-ODNs) infused intracerebrally in key loci to knockdown the expression of the plasticity-associated gene *zif268* normally induced by memory reactivation (Lee et al., 2005). Pavlovian cue-drug memories, linking environmental stimuli to a drug high, reconsolidate (Milton et al., 2008, Sanchez et al., 2010, Theberge et al., 2010, Barak et al., 2013); but whether instrumental *habit* memories can also be specifically targeted for disruption is unclear.

Until recently, instrumental memories were thought not to reconsolidate, as protein synthesis inhibition did not produce reactivation-dependent amnesia (Hernandez and Kelley, 2004; for review see Vousden and Milton, 2017). However, early studies did not take into account that instrumental behavior can be supported by either goal-directed (‘action-outcome’, A-O) or habitual (‘stimulus-response’, S-R) associations. These associations form in parallel (Dickinson, 1985) and are psychologically and neurobiologically dissociable. The A-O association is mediated by the posterior dorsomedial striatum (pDMS) while the automaticity of responding, as it becomes an S-R habit, progressively engages the anterior dorsolateral striatum (aDLS) (Haber, 2003, Belin and Everitt, 2008, Zapata et al., 2010, Murray et al., 2012) and requires an intact aDLS and posterior dorsolateral striatum (pDLS) (Packard and McGaugh, 1996, Yin et al., 2004). Although some data indicated that instrumental memories are robust because they do *not* undergo reconsolidation (Hernandez and Kelley, 2004), other studies have challenged this, showing that systemic NMDAR antagonism can disrupt instrumental memory reconsolidation under specific conditions (Exton-McGuinness et al., 2014).

Determining whether instrumental responding is goal-directed or habitual can be achieved through outcome devaluation (Dickinson, 1985) and contingency degradation (Hammond, 1980). A related method, first employed by Tolman et al. (1946) and adapted by Packard and McGaugh (1996), uses a modified T-maze task, which produces a different behavioral outcome depending upon which association is retrieved during a probe test. Briefly, animals are trained to run to a specific rewarded location in a T-maze. Animals can retrieve the reward either by using extramaze (allocentric) cues to produce a spatial ‘place’ representation of the goal, or by encoding the motion (egocentric) cues required to reach the goal (e.g. ‘turn left’). In a probe test, animals start opposite the original starting location. Therefore, an A-O response leads to ‘place’ learners correctly choosing the previously baited arm on the probe test, whereas ‘response’ learners employ the body turns used in training (i.e. respond incorrectly/S-R).

Inactivation studies have shown the hippocampus to be necessary for expression of the ‘place’ memory whereas the dorsolateral striatum supports the ‘response’ memory in this T-Maze task (Packard and McGaugh, 1996). Of particular interest, from a reconsolidation perspective, is the finding that instrumental training can increase striatal expression of *zif268,* and that after extensive training it remains elevated only in lateral striatal regions (Maroteaux et al., 2014). This is consistent with our preliminary data, showing that Zif268 was upregulated in the posterior (but not anterior) dorsolateral striatum (pDLS) of response learners in the T-Maze task (Milton and Everitt, 2012). As Zif268 is critical for appetitive pavlovian memory reconsolidation (Lee et al., 2006), we analyzed the expression of Zif268 after extended training in the T-Maze task and investigated whether *zif268* knockdown in the pDLS using ASO-ODNs during memory reactivation would disrupt the subsequent expression and persistence of a response memory.

## Experimental procedures

### Subjects

Subjects were 101 male Lister-Hooded rats (Charles River, Bicester, UK), weighing 250 g at the start of the experiment, that were housed in pairs in a vivarium maintained at 21 °C, on a reversed light–dark cycle (lights on at 1900 h). Water was available *ad libitum* except during behavioral training and testing sessions, and the animals were food-restricted at 85–90% of their free-feeding weight, being fed after behavioral procedures each day. Weights were monitored thrice-weekly. All procedures were conducted in accordance with the UK Animals (Scientific Procedures) Act 1986.

### Behavioral apparatus

Each animal was tested individually on a plus maze with four arms of 50 cm long and 15 cm wide, at a height of 50 cm from the floor, with raised sides of 4 cm. One arm of the plus maze, opposite to the start arm, was occluded by a white Perspex door, converting the apparatus into a T-maze. The maze was situated in a room with many external cues located around the maze, and these cues remained the same throughout training and testing of each batch of animals.

### Surgery

Rats were anesthetized with intramuscular injections of a mixture of ketamine (Ketaset; Henry Schein, Dumfries, Scotland, 0.1 ml/100 g body weight) and xylazine (Rompun; Henry Schein, 0.05 ml/100 g body weight). Each rat was placed into a stereotaxic frame (David Kopf, USA) and implanted with guide cannulae (24-gauge, 11-mm; Cooper’s Needleworks) targeting the pDLS, using the following co-ordinates (mm): AP −0.4 mm, ML ±4.0 mm (from bregma), DV −3.8 mm (from the skull surface). Wire stylets (Cooper’s Needleworks) were inserted into the guide cannulae to maintain patency. Rats were allowed at least 7 days of recovery from surgery before behavioral procedures began.

### Behavioral procedures

Behavioral procedures were adapted from those described by Packard and McGaugh (1996). Prior to training, each rat received two days of habituation to the T-maze, and to the sucrose pellet reward (Noyes 45-mg pellets, Sandown Scientific, UK). Each rat was placed in the maze for 5 min and allowed to freely explore, and following return to the home room was given 10 sucrose pellets in the home cage.

During behavioral training, rats were removed from their home cages and placed in a holding cage prior to the start of the trial. At the start of the trial each rat was placed in the ‘start’ arm, which was the same for each rat, and the timer started. One arm of the T-maze was baited with a single sucrose pellet; the rewarded arm was counterbalanced between rats, but remained the same throughout training for each rat. Each rat was given four trials on the maze each day, with trials separated by a 30-s intertrial interval (ITI) during which the rat was placed back into the holding cage. If the rat entered the incorrect arm during training, it was allowed to remain in the maze until the correct arm was chosen, or a predetermined ‘time-out’ of 120 s was reached. The experimenter remained in the room throughout testing, manually recording the latency to retrieve the pellet and the number of incorrect responses on each trial. The experimenter stood in the same position, behind the start arm, during all trials. On the last two days of training, the rats were habituated to the intracerebral infusion procedure at least once.

Following the completion of training, the rats underwent a memory reactivation session, designed as a ‘probe’ session. In this probe session, the Perspex occluder was moved to the original start arm, so that the rats started the probe test in the arm *opposite* the original start arm, though the maze itself remained in the same position relative to the rest of the objects within the room. During the probe test, no sucrose pellets were available, and the rats were only allowed to enter one arm, on a single trial. The experimenter remained in the room throughout the probe test, recording arm choice and the latency to reach the end of the arm. The use of a different starting position in the maze was to allow determination of whether the rats were using a ‘place’ representation of the pellet location, or a ‘response’ representation, as previously described (Packard and McGaugh, 1996). Rats using the ‘place’ representation to guide behavior chose the same spatial location as in training, turning in the *opposite* direction to training; rats using the ‘response’ representation to guide behavior would make the *same* response, and so move into the arm *opposite* the trained arm, away from the environmental cues associated with reinforcement during training. 90 min prior to this behavioral session, the rats received bilateral infusions of either *zif268* antisense (ASO) or as a control missense (MSO) oligodeoxynucleotides into the pDLS. A ‘delayed infusion’ control group received an ASO infusion 6 hours following the probe test, as it has been shown in previous studies that levels of Zif268 return to baseline at 4–6 hours after retrieval (Lee et al., 2004, Milton and Everitt, 2012). Animals were allocated to groups after counterbalancing for performance (measured by latency and number of incorrect trials) and reward location during training.

A probe test was conducted 72 hours following memory reactivation (‘Test 1’). In order to assess the persistence of any deficit, subsequent tests were conducted 1 week (‘Test 2’) and 1 month (‘Test 3’) following reactivation.

### Drug preparation and intracerebral microinfusions

Oligodeoxynucleotides (ODNs) were PAGE-purified phosphorothioate end-capped 18-mer sequences resuspended in sterile phosphate-buffered saline (PBS) at a concentration of 2 nmol/μl (Zif268 antisense ODN: 5′-GGT AGT TGT CCA TGG TGG-3; Zif268 scrambled missense ODN: 5′-GTG TTC GGT AGG GTG TCA-3′, Alta Bioscience). Based on our previous work, the ASO-ODNs were expected to knock down Zif268 expression acutely by approximately 60%, with expression levels recovering 24 hours later (Lee et al., 2005).

Infusions were carried out using a syringe pump and 5-μl Hamilton syringes, connected to injectors (28 gauge, projecting 1 mm beyond the guide cannulae) by polyethylene tubing. Infusions of ODNs (1.0 μl/side, 0.125 μl/min) took place 90 min prior to the memory reactivation session. Injectors were inserted 30 s prior to the start of the infusion, and remained in place for 60 s after the end of the infusion, to allow diffusion of the solution away from the infusion site. Rats were habituated to the infusion procedure at least once on the two days prior to memory reactivation.

### Histological assessment of cannulae placements

After the completion of testing, the rats were killed with an overdose of sodium pentobarbital (2.0 ml per animal of Dolethal, Rhone Merieux, UK) before undergoing perfusion-fixation with 0.01 M PBS, followed by 4% paraformaldehyde (PFA). The brains were removed and stored in 4% PFA, before being transferred to 20% sucrose for at least 8 hours prior to sectioning. The brains were coronally sectioned at 60 μm around the guide cannulae and stained using Cresyl Violet. The cannulae placements were subsequently verified by eye using a Leitz DMR-R microscope (Leica, Milton Keynes, UK).

### Sample preparation and Western blotting

Either 2 hours or 6 hours post reactivation, animals were sacrificed by CO_2_ asphyxiation and the brains rapidly removed, frozen on dry ice and subsequently stored at −80 °C. Samples from the basolateral amygdala (BLA), hippocampus, nucleus accumbens, anterior and posterior dorsolateral striatum and anterior and posterior dorsomedial striatum were microdissected using a 0.99-mm-diameter punching tool from 150-μm-thick frozen brain sections (see Fig. 5). The punched tissue from each animal was briefly sonicated in 200 μl of cold lysis buffer (0.32 M Sucrose, 20 mM Tris, 1 mM EDTA, 1 μg/ml Pepstatin A, 10 μg/ml leupeptin, 0.5 mM PMSF, and 10 μg/ml aprotinin) and centrifuged at 5000 rpm for 5 min at 4 °C. The supernatant was transferred to a clean tube and stored at −20 °C. The protein content was quantified using a spectrophotometer (Nanodrop). 5–10 μg of samples were loaded and separated using a 10% SDS–PAGE and electrotransferred onto a nitrocellulose membrane (Thermofisher Scientific, UK). Blots were probed with the following antibodies which were tested to deliver a linear relationship between the amounts of loaded protein in the blot and signal intensity: rabbit anti-Egr1 (Zif268, 1:300; Santa Cruz); mouse anti-beta actin (1:6000; Abcam); goat anti-rabbit-HRP (1:2500; Sigma Aldrich); and rabbit anti-mouse-HRP (1:5000; Sigma Aldrich) diluted in 1% non-fat dried milk (Marvel) in Tris-buffered saline solution containing 0.25% of Tween-20. A chemiluminescent signal was induced using an enhanced chemiluminescent reagent (GE Healthcare), and images were captured using a CCD camera (ChemiDoc-It, UVP). Samples were run at least in duplicates. Signal analysis and quantification were performed using ImageJ software (version 1.49 m, National Institutes of Health). The optical density (OD) of the bands of interest was measured, and normalized to OD of the loading control (β-actin).

### Statistical analyses

Data are presented as mean ± SEM, unless otherwise stated. Western blotting data were analyzed using a one-way analysis of variance (ANOVA) with Dunnett’s test for post hoc comparisons. Behavioral differences between groups (ODN infusion and timepoint of tissue collection) during training on the T-Maze were analyzed by repeated-measures ANOVA with Day as a within-subject factor and Group as the between-subject factor. Where the assumption of sphericity was not satisfied by Mauchly’s test, a Greenhouse-Geisser correction was applied. The categorical arm choice data collected at memory reactivation and tests were analyzed using a Chi-squared comparison. For Western Blotting, 2H time points for both strategies, when Zif268 is highly expressed, were compared to the 6H time point of the place group by analysis of variance and post-hoc comparison of means by Dunnett’s test. Tests were carried out using GraphPad Prism 4.0 software (GraphPad Software Inc., San Diego, CA) and IBM SPSS Statistics software 22 (IBM, UK). The significance level was set at *p* < 0.05.

## Results

### Behavioral performance

**Fig. 1:**
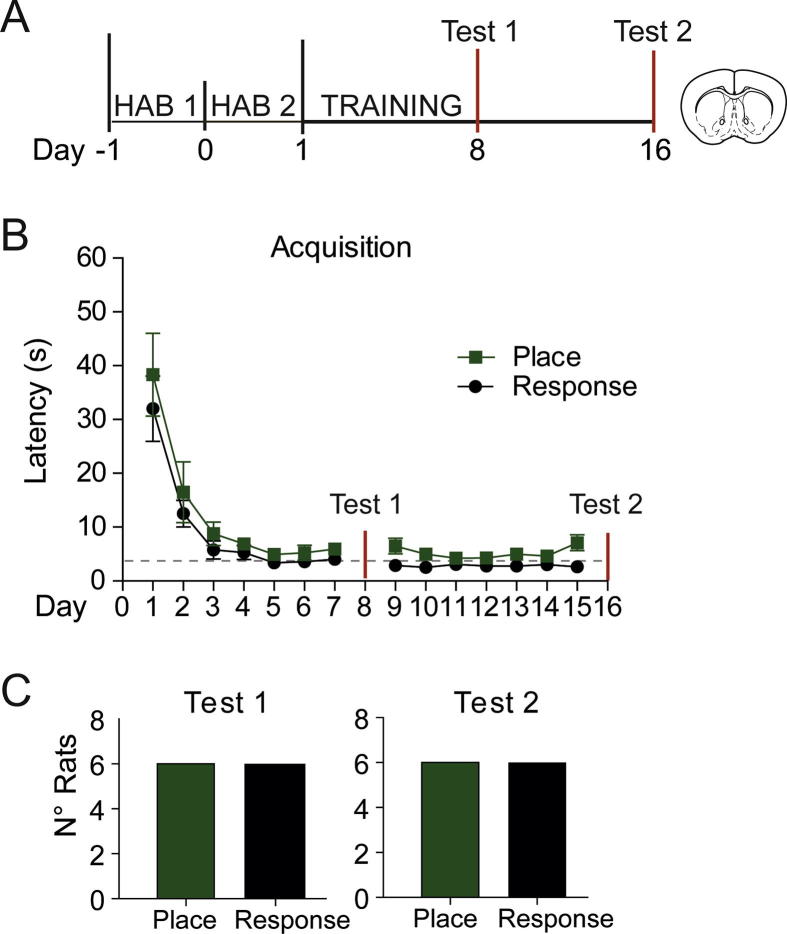
12 uncannulated rats were trained to collect reward pellets from a specific location on the T-maze, with probe tests conducted on Days 8 and 16 to determine whether they were using a place or response strategy. (A) Schematic of protocol. (HAB = habituation to the T-maze apparatus). (B) Latency to collect the reward pellet decreased throughout training, with no difference in latency across the groups by choice of strategy used at test. The gray dotted line represents the average latency of the last 7 days, where performance plateaued (4.02 s). (C) After 7 days of training the proportion of animals using a place or habit response strategy was equivalent. 7 days later the strategy proportions remained the same, even though at an individual level many animals used a different strategy at the second test.

*Experiment 1:* Rats (total *n* = 12) were trained on a reinforced T-Maze task as described previously to establish a set of place or response strategy-using animals (Packard and McGaugh, 1996). All rats learned which arm was baited with sucrose and the latency to retrieve it plateaued by Day 5, [*F*(13,130) = 31.8, *p* < 0.001, *ɲ*^2^ = 0.76], with no difference between the groups [*F*(1,10) = 2.31, *p* = 0.997] (Fig. 1B). Contrary to previously reported findings, the proportion of rats using a place or response strategy after 8 days of training was equivalent at the first probe test (Test 1). The proportion remained constant also at Day 16 (Test 2), despite 8 out of 12 rats switching strategy from the last probe test (Fig. 1C).

**Fig. 2:**
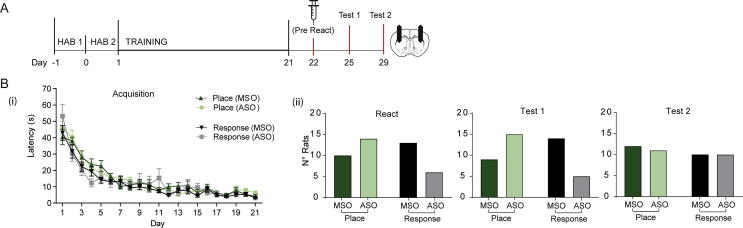
The effects of Zif268 knockdown in the pDLS on the reconsolidation of the memory underlying a response strategy was assessed in 43 cannulated rats previously trained on the T-maze. (A) Rats were implanted with cannulae targeting the DLS and underwent training in the T-Maze as before. (B) (i): Latencies to collect the reward decreased during training, with no difference between the prospective MSO or ASO groups. (ii) Infusion of the ASO-ODNs (Day 22) did not affect the latency to reach the reward location during the reactivation session. A Chi-square test of independence was performed to examine the relationship between ODN administration and strategy at Reactivation. The relationship between these variables was not significant. These data show that acute knockdown of Zif268 did not influence the strategy selected to perform the task. The effects of Zif268 knockdown at reactivation were probed 72 h later (Test 1). There was a significant association between ODN administration and the type of strategy used at test. However, in post hoc comparison, when ASO was administered the standardized residual approached but did not reach significance for a habit strategy (*z* = −1.3). Animals were tested one week after reactivation (Test 2) and the proportions of response to place strategy were unaffected by prior ODN administration.

**Fig. 3:**
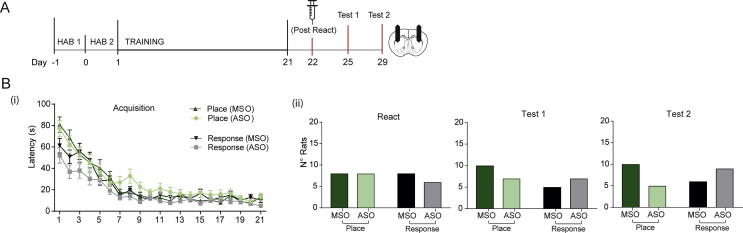
There was no effect of Zif268 knockdown in the pDLS on subsequent strategy choice in 30 rats when ODNs were administered outside the reconsolidation window. (A) Protocol described as before, except that animals were administered a delayed infusion of the ODN (MSO or ASO) 6 h after reactivation (outside the reconsolidation window) as a control. (B) (i): Latencies to collect the reward decreased with training, with no difference between the prospective MSO and ASO groups. (ii): The infusion of ODN 6 h after reactivation had no effect on the subsequent strategy used at the long-term memory tests 72 h and 1 week after reactivation.

*Experiment 2:* It was previously reported that Zif268 expression was increased specifically in the pDLS of response rats (Milton and Everitt, 2012). We predicted that a knockdown of Zif268 expression by using ASO-ODNs in the pDLS would disrupt the reconsolidation of the response memory after reactivation versus the control MSO-infused animals. In order to test this hypothesis rats (*n* = 73) were implanted with cannulae aimed at the pDLS and then trained as before. One squad (*n* = 43) were tested for the effects of ODN infusion before reactivation and the other (*n* = 30) 6 hours post reactivation, as a control. For the first squad, latencies to collect the reward decreased with training [*F*(4.43,173) = 54.9, *p* < 0.001, *ɲ*^2^ = 0.59], with no difference between the groups [*F*(1,39) = 0.751, *p* = 0.392]. However, cannulation of the animals resulted in a slower acquisition curve (data not shown) for the task when compared to the uncannulated animals in the first experiment and therefore training was extended to 21 days. On Day 22 the first squad (*n* = 43) were first infused with ASO or MSO 1 h before being introduced to the inverted maze without reward, to reactivate the memory (React). There was no significant effect of ODN on expression of the response or place memory during the Reactivation session [*X*^2^ (1, *N* = 43) = 3.05, *p* = 0.081] (Fig. 2B ii), although more animals were in the ‘place’ group overall. 72 hours later (Test 1), the subsequent effect of ASO treatment on memory reconsolidation was tested. A significant decrease in the proportion of animals using a response memory was observed, [*X*^2^ (1, *N* = 43) = 5.58, *p* = 0.018]. However, in post hoc comparison, when ASO was administered, the standardized residual approached but did not reach significance for a habit strategy (*z* = −1.3). The odds ratio determined that the odds of using a response strategy and having received MSO-ODN were 4.67 times higher than if the rat was treated with ASO-ODN (Fig. 2B ii). However, this effect did not persist 7 days later (Test 2) as the proportions were equivalent across the groups [*X*^2^ (1, *N* = 43) = 0.02, *p* > 0.05]. As a control, ODN administration 6 h post-reactivation (*n* = 30) did not affect responding in subsequent tests (Fig. 3B ii). Overall, these results indicate that knockdown of *zif268* in the pDLS alone does not permanently disrupt a response memory.

**Fig. 4:**
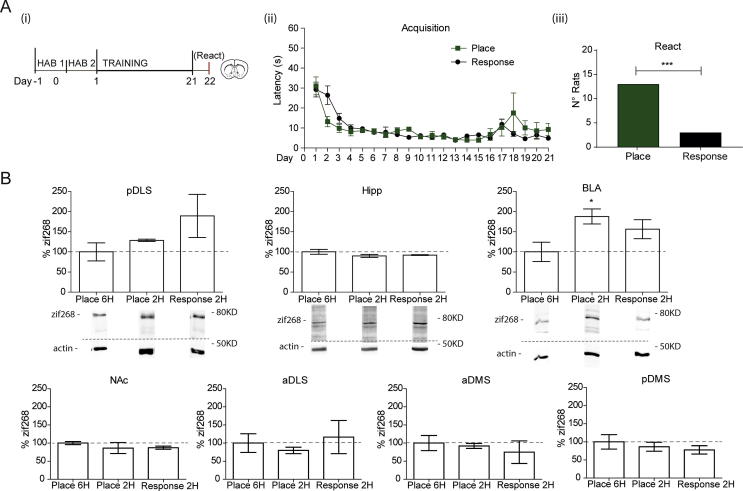
16 rats underwent a memory reactivation session after 21 days of training on the T-maze, before brains were harvested for assessment of Zif268 expression. (A) (i): The rate of acquisition followed that of Experiment 1, although training was extended to 21 days to compare to the cannulated animals in Experiment 2. (ii): Latency to collect the reward decreased with training with no effect of strategy used at Test 1. (ii) Extended training of uncannulated rats resulted in a significant shift in the proportion of animals using a place strategy, ^***^p < 0.001. (B) In samples taken from the pDLS, a non-significant increase in the expression of Zif268 relative to the 6 hours control group was seen in the rats using a response strategy, although the number of animals here is too small to draw a strong conclusion for the response group. The use of a place strategy did not appear to increase Zif268 expression in the hippocampus. The levels of Zif268 were not altered in the hippocampus by use of either strategy. Interestingly, there was an apparent increase in Zif268 levels in the BLA regardless of the strategy used at test. There was no significant difference in Zif268 levels after reactivation in the NAc, aDLS, aDMS nor pDMS. Bars are mean per group (Place 6H *n* = 5, Place 2H *n* = 6, Habit 2H *n* = 2) +/− SEM; images are representative blots.

*Experiment 3:* Zif268 is a key plasticity protein expressed in response to reactivation of various forms of memory across limbic regions. We therefore anticipated to detect changes in Zif268 levels across limbic regions depending on strategy used at reactivation. To investigate this we trained another group of rats (total *n* = 16) for 21 days in order to compare with the previous Experiment 2 (Fig. 4A i). Importantly, the rats were not cannulated and the task was acquired quickly as latency decreased significantly over training [*F*(20,280) = 10.3, *p* < 0.001, *ɲ*^2^ = 0.42] at a rate very close to that found in Experiment 1 (Fig. 1B). It was observed that a significant majority of animals used a place strategy at reactivation: *χ*2 (1, *N* = 16) = 16, *p* < 0.001 (Fig. 4A iii). This is contrary to predictions, based on previous data, that with extended training a response symptomatic of habit will form and dominate behavior (Packard and McGaugh, 1996).

**Fig. 5:**
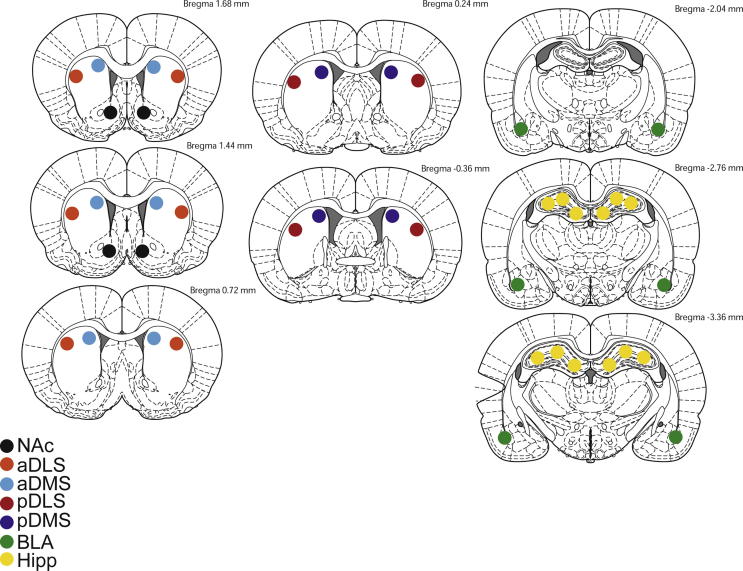
Illustration of location, start and end points for tissue collection punches from the regions analyzed for Zif268 expression by western blotting.

### Zif268 expression

Zif268 expression is induced by reactivation of many forms of memory (Veyrac et al., 2014). We measured Zif268 expression after memory reactivation across brain regions recruited by the T-Maze task. Animals were randomly allocated to a 2-hour (when Zif268 levels should be elevated) or 6-hour time-point (control, when Zif268 levels have decreased) for sacrifice. As only three animals used a response strategy, one was allocated to the 6-hour group for comparison but was not included for statistical analyses. Based on previous findings, it was predicted that the levels of Zif268 should increase in the pDLS following reactivation of a response memory. Due to only a small proportion of animals using a response strategy after 21 days of training, the expected increase was visibly detected but not statistically significant (Fig. 4B). In contrast, there was no change in Zif268 expression in the group using a place strategy. However, despite the requirement of hippocampal activity for the expression of the place strategy (Packard and McGaugh, 1996), Zif268 expression did not increase in the hippocampi of animals that had used this strategy at reactivation (Fig. 4B). Interestingly, a significant increase of Zif268 expression was detected in the BLA sample for the place 2H group by ANOVA [*F*(2,12) = 4.240, *p* < 0.05] using group as the factor, revealed by a post-hoc Dunnett’s Test versus the control group (Fig. 4B). The other striatal regions (namely the NAc, aDLS, aDMS and pDMS) did not have altered Zif268 levels (Fig. 4B).

## Discussion

In this study, we investigated the requirement of the immediate early gene *zif268* in the pDLS for the reconsolidation of an S-R memory. Our previous, preliminary data (Milton and Everitt, 2010) had indicated that animals, trained on a T-maze and using a S-R strategy in a probe test, had increased levels of Zif268 expression selectively in the pDLS. We therefore investigated whether Zif268 expression in this locus was causally involved in the reconsolidation of the habit-like memory that underlies S-R responding. Contrary to our predictions and previous work (Packard and McGaugh, 1996), we found that: (i) extended training did not result in a preponderance of animals using an S-R strategy; (ii) knockdown of Zif268 in the pDLS prior to memory reactivation altered the proportion of animals using an S-R strategy 72 hours after reactivation but not at the subsequent test (7 days after reactivation), and; (iii) there were no changes in Zif268 expression in the hippocampus of animals using an place strategy during a probe test. Our previous finding, that Zif268 increased in the pDLS of animals using an S-R strategy during a probe test, was replicated but likely underpowered. Finally, exploratory analyses revealed an increase in Zif268 expression in the basolateral amygdala 2 hours after the probe test, irrespective of the response strategy used.

It had been previously reported that following 14 days of training, approximately 80% of animals use the response strategy in a probe test (Packard and McGaugh, 1996). By contrast, we saw approximately equal numbers using the ‘place’ and ‘response’ strategies. This seems to contradict predictions that with extended training a habit-like response should form. Furthermore, we found that extensive training (21 days) led to the majority of rats using a place strategy. A number of factors can influence response strategy including environment complexity, rat strain, motivation and anxiety (Scharlock, 1955, Restle, 1957, Asem and Holland, 2013). The rate of task acquisition, i.e. latency to obtain reward, in cannulated animals in the present experiments was slower than in uncannulated rats and took longer to plateau as compared to uncannulated animals. There is extensive evidence that stress, even vehicle injection, may promote the use of response strategies in both rodents (Schwabe et al., 2010) and humans (Schwabe, 2013). The uncannulated animals may have been exposed to fewer stressful events, which may be related to their faster acquisition of the task and hence the high proportions of place responders at Day 21.

Zif268 was targeted in this study due to its established role in memory reconsolidation and our preliminary observation that it was increased in the pDLS at reactivation. Zif268, along with other IEGs (e.g. *c-fos*) are commonly used as markers of synaptic activity as their shortlived expression is tightly linked with activity and, in particular, glutamate receptor activity. However, the direct targets of this transcription factor that mediate its effects on memory and learning are still unknown. This is despite some signaling mechanisms being established by the study of pathological systems, although these may not directly apply to the *in vivo* role of Zif268 in memory (Veyrac et al., 2014). In our previous work, *zif268* ASO knocked down Zif268 expression by approximately 60%, when compared to the MSO (Lee et al., 2006). Herein, knockdown of Zif268 expression in the pDLS prior to memory reactivation did not alter the response strategy chosen during the probe trial (the memory reactivation session), and although it reduced the likelihood of rats using the ‘response’ strategy shortly (72H) after the manipulation, this effect was transient and did not persist to subsequent tests. In a previous study in mice, Zif268 expression remained elevated in the pDLS, but not DMS, after 5 days of a food-rewarded operant task (Maroteaux et al., 2014). However, a similar study in rats showed increased Zif268 mRNA in the aDMS and aDLS after limited training, but after extensive action-outcome pairings there was a non-significant decrease in Zif268 mRNA (Hernandez et al., 2006). On the other hand Homer1a remained elevated, although the authors did not test if behavior had become habitual in nature. Different stages of memory training may therefore preferentially recruit different immediate early genes. Here, targeting of *zif268* in the pDLS alone may not be sufficient to disrupt the plasticity of the reactivated response memory.

Based on previous data (Packard and McGaugh, 1996) showing that inactivation of the hippocampus biased animals away from using a place strategy, we predicted that the hippocampus would be required for the reactivation as well as retrieval of the place strategy, and that this would be correlated with the expression of Zif268. However, we did not observe an increase in Zif268 in the hippocampus of rats using the ‘place’ strategy at test. This is in agreement with a previous report using Zif268 immunohistochemistry after explicit place or response training in the T-maze in which neither place nor response testing induced hippocampal Zif268 expression above control levels, whereas c-Fos levels differed depending on the strategy used (Gill et al., 2007). Therefore, these data together indicate that the hippocampus may recruit a variety of IEGs at reactivation and knockdown of *zif268* in the pDLS does not prevent the expression or restabilization of the T-Maze place memory.

Regardless of the strategy used, we observed increased levels of Zif268 in the BLA. In both groups a common feature of the reactivation procedure is a violation of expectation, as they should predict to receive the sucrose reward at the end of the arm and in both cases it was absent. This may suggest this activation of Zif268 in the BLA in both groups is linked to a negative prediction error signal. In the NAc, which is well-documented as receiving information about positive prediction error signals (Schultz et al., 1997), there was no change in Zif268 expression across groups (Fig. 4B). While there is some evidence that encoding of reward absence in extinction recruits amygdala neurons and therefore may induce plasticity-related gene expression (Tye et al., 2010), there is no evidence that the probe tests are sufficient to induce extinction learning. There is a theoretical negative prediction error in both instances, which could engage either reconsolidation or extinction, but whether this causally induces Zif268 expression remains unclear.

The pDLS was targeted in this study based on the observation of a specific increase in Zif268 (Milton and Everitt, 2012, this present study). Excitotoxic combined lesion of the aDLS and the pDLS showed that these regions are involved in S-R responding (Yin et al., 2004); similarly inactivation of the pDLS prevented rats from using a S-R strategy at test (Packard and McGaugh, 1996). We failed to detect any increase in Zif268 in the aDLS, which is perhaps surprising given the known involvement of this area in the acquisition and expression of stimulus–response habits (Zapata et al., 2010, Murray et al., 2012). However, as any distinct role of the pDLS was not directly investigated in those studies and the tasks were different to that used in the present study, it is difficult directly to compare across them. Whether restabilization of a response memory can be disrupted by *zif268* knockdown in the aDLS merits further investigation.

## Conclusion

Specific knockdown of Zif268 in the pDLS alone appears not to be necessary for the reconsolidation of the habit-like memory underlying an S-R strategy on the T-maze. Whether knockdown of Zif268 in other structures engaged by memory reactivation – such as the basolateral amygdala and/or aDLS – would produce longer lasting changes in response strategy remains an open question.
